# Clinical effect of application of nursing concept of rehabilitation surgery for improvement of quality of postoperative recovery in orthopedics

**DOI:** 10.1186/s13018-021-02610-3

**Published:** 2021-07-30

**Authors:** Hong Lv, Ning Yang

**Affiliations:** Inner Mongolia Sports Hospital, No.2 South Tongdao Road, Huimin District, Hohhot, 010030 Inner Mongolia Autonomous Region China

**Keywords:** Concept of accelerated rehabilitation surgery, Orthopedics, Perioperative period, Postoperative recovery, Quality of nursing, Occurrence rate of complications

## Abstract

**Objective:**

To analyze the application of concept nursing of accelerated rehabilitation surgery in orthopedic postoperative recovery.

**Methods:**

A total of 120 patients who received orthopedic surgery were divided into the control group undergoing routine orthopedic nursing and the observation group undergoing the concept of accelerated rehabilitation surgery nursing.

**Results:**

Patients in the observation group had shorter in-bed activity time and out-of-bed activity time, average time of hospital stay, and lower total treatment costs. The incidence of incision infection, respiratory system infection, digestive tract infection, urinary tract infection, deep vein thrombosis, and other complications in the observation group was much lower. The recovery scores of joint function in the observation group at 1, 3, 6, and 12 months after the operation were all better, and the recovery rate of joint function within 1 year after the operation was higher.

**Conclusion:**

Following the concept of accelerated rehabilitation surgery nursing during the perioperative period can improve the quality of postoperative orthopedic recovery.

## Introduction

Orthopedics is one of the key clinical departments of general hospitals at all levels, which not only studies the anatomy, physiology, and pathology of the human skeletal muscle system, but also deeply explores the application effect of drugs, surgery, and physics in maintaining the normal shape and function of this system [1]. In recent years, with the time change and social development, the orthopedic injury spectrum has also gradually changed. For example, orthopedic diseases such as bone and joint tuberculosis, osteomyelitis, and poliomyelitis have decreased, while traumatic fractures caused by traffic accidents or other causes have increased dramatically [2]. In terms of the clinical treatment of fracture patients, the corresponding surgical plan can achieve a better effect, helping the reduction of fracture and functional recovery, but the trauma of surgery can still have a certain impact on the physiological aspects of patients. If effective nursing programs cannot be coordinated in the perioperative period, patients of orthopedic surgery tend to suffer from infection, thrombosis, and other complications, which will reduce the surgical effect and hinder postoperative recovery [3]. The concept of accelerated rehabilitation surgery is a perioperative nursing concept of surgical patients first proposed by Danish surgeon Kehlet, which has been widely applied in orthopedic clinics at present [4]. The nursing mode based on the concept of accelerated rehabilitation surgery mainly refers to the adjustment and intervention of nurses to physiology, psychology, and other aspects of patients by using the comprehensive knowledge of clinical medicine during the operation, so as to reduce the stimulation of surgery, prevent related complications, and promote postoperative recovery [5]. We assumed that the concept of accelerated rehabilitation surgery could improve postoperative orthopedic recovery. This study aims to explore the effect and clinical value of following the concept of accelerated rehabilitation surgery to improve the quality of postoperative orthopedic recovery.

## Materials and methods

### General materials

This study has been submitted to the Ethics Committee for approval, and the patients have been informed of the agreement. A total of 120 patients with traumatic limb fractures who received orthopedic surgery in our hospital from February 2017 to February 2019 were randomly selected. They were divided into two groups on average according to the time of admission, with 60 cases in the control group and 60 cases in the observation group.

#### Inclusion criteria

The following are the inclusion criteria: in clinical practice, traumatic limb fractures were confirmed by X-ray, CT, and other imaging examinations, which were in line with the indications of orthopedic surgery [6]; there were no movement difficulties before fracture and can walk independently; the time from injury to admission did not exceed 10 days; older than 18 years; normal cognitive function and effective communication; and signed the informed consent form before enrollment.

#### Exclusion criteria

The following are the exclusion criteria: patients with traumatic limb fracture combined with systemic fracture; patients with acute critical condition of the heart, brain, kidney, and other important organs; patients with limb movement dysfunction before the operation; patients who are unable to cooperate with the investigator due to mental illness, unclear consciousness, and cognitive dysfunction; follow-up falls off; and patients whose clinical data is incomplete.

### Research methods

#### Control group

In the control group, routine orthopedic nursing was applied without special intervention during the perioperative period. The contents include the following: the responsible nurse comprehensively grasps the basic condition of the patient and records the treatment situation in detail, assisted the patient to complete the basic preoperative examinations, ordered the patient to abstain from drinking for 4 h and fasting for 8–12 h, and closely monitored the perioperative vital signs; during the operation, according to the type of anesthesia, the patient should be assisted to place the body in a reasonable position, provide oxygen support, establish venous channels, and insert catheters and other basic nursing work, and the nursing staff should cooperate with the competent physician to complete the treatment measures, doctor’s advice, and nursing; and during the period of hospitalization, the nurses carried out the universal education of disease knowledge, guided them to learn self-care skills, and improve their daily diet and other living habits.

#### Observation group

The concept of accelerated rehabilitation surgery nursing for patients in the observation group during the perioperative period was followed. Based on the control group, measures were taken: orthopedic medical personnel were organized to set up a nursing management group for the concept of accelerated rehabilitation surgery, with the head nurse as the group leader and a senior nurse with 5 years of clinical experience as the supervisor. Since the patient was admitted to the hospital, the nursing staff comprehensively collected and sorted out the basic condition, previous medical history, living habits, and other data of the patient and formulated the nursing plan and implementation plan of the concept of rehabilitation surgery combined with the reality. Following the concept of accelerated rehabilitation surgery, the patient can relax the preoperative abstinence from drinking and fasting, receive non-solid food at 6 h before surgery, and take 500-ml glucose solution at 4 h before surgery with a concentration of 10%; the nursing staff pay close attention to the psychological dynamic changes of patients, provide timely preoperative counseling to eliminate the tension, anxiety, and other adverse emotions of patients, and help patients increase confidence by introducing successful cases of surgery; during indwelling catheter, observe whether there is urinary retention after the operation. If there is urinary retention, then insert the catheter. Pay attention to the fluid flow, color, and nature of the catheter and remove at the appropriate time. If the patient has no obvious symptoms of discomfort, drinking water can be started at 4 h after the surgery, and liquid food can be introduced at 12 h after surgery, give internal controlled analgesia to alleviate the symptoms of limb pain within 2 days after the surgery; conduct individualized health education to the patients, and it was important to inform them that early rehabilitation exercise in orthopedic surgery could promote the recovery of limb joint function. Combine with the specific postoperative recovery situation of the patient, instruct them to conduct activities in and under the bed, keep activity level and intensity gradually progressive, and assist massage technique to prevent deep vein thrombosis; observe the changes of perioperative body temperature. If the body temperature within 3 days after the operation is within the normal range, the analgesic effect during the hospitalization is good, and if there are no infection-like complications, the patient can eat smoothly and move freely, then the discharge standard is considered to be met. Generally, the patient can be discharged within 2 weeks after the operation. The patient was given discharge guidance, and the indicators related to the vital signs, limb function, and psychological state were re-evaluated. Specific joint function recovery training programs were formulated, and nurses and patients or their families were contacted by phone and WeChat to achieve postoperative rehabilitation guidance and regular follow-up.

### Evaluation index

1. The in-bed and out-of-bed activity time, average hospital stay time, and total treatment costs of the two groups of patients were recorded.
2. The incidence of complications in the two groups within 4 weeks after surgery was observed and counted, including incision infection, respiratory system infection, digestive tract infection, urinary tract infection, and deep vein thrombosis.
3. The follow-up period for 1 year; the two groups of patients with postoperative 1 month, 3 months, 6 months, and 12 months when limb joint function recovery rate; evaluation content involves the scope of joint activities, joint function, degree of pain, deformity, etc.; out of 100 points, the higher the score that joint function recovery, the better; in addition, the score ≥ 60 points 1 year after the operation was included in the excellent and good rate of joint functional recovery for comparative analysis.
4. The nursing satisfaction scale of the department of orthopedics of our hospital was adopted to investigate the satisfaction degree of the two groups of patients with clinical nursing, and the assessment content involved nursing service attitude, nursing professional ability, nursing details implementation, and nursing experience. The full score is 100, the score < 60 is “not satisfied,” 60-80 is “basically satisfied,” and > 80 is “very satisfied.” A higher score means higher patient’s satisfaction with the nursing. Compare the overall satisfaction rate (the sum of the extraordinary satisfaction rate and the basic satisfaction rate).

### Statistical analysis

The patient data and related data of the study results were processed by the statistical software SPSS 20.0. The mean ± standard deviation (*x̄* ± *s*) was used to represent the measurement data, and the rate (%) was used to represent the counting data. The test values between the groups were *t* and *x*^2^. *P* < 0.05 was set as a statistically significant difference.

## Results

### Basic characteristics

A total of 120 patients with closed traumatic limb fractures who received orthopedic surgery in our hospital from February 2017 to February 2019 were randomly selected. They were divided into two groups on average according to the time of admission, with 60 cases in the control group and 60 cases in the observation group. In the control group, there were 38 males (63.33%) and 22 females (36.67%), aged 18–75 years, with an average age of (38.54 ± 6.72) years. The time from fracture to admission was 3 h to 10 days, and the mean course of the disease was 4.22 ± 2.30 days, 25 cases of upper limb fracture (41.67%) and 35 cases of lower limb fracture (58.33%). In the observation group, there were 39 males (65.00%) and 21 females (35.00%), aged 18–72 years, with an average age of 37.98 ± 6.84 years. The time from fracture to admission was 3 h to 10 days, and the mean course of the disease was 4.45 ± 2.36 days, 24 cases of upper limb fracture (40.00%) and 36 cases of lower limb fracture (60.00%). There were no statistically significant differences in gender composition, mean age, duration of disease, and general materials of disease between the two groups, and a comparative study can be conducted (*P* > 0.05).

### Comparison of in-bed and out-of-bed activities, hospital stay, and total cost of treatment between the two groups

Compared with the control group, patients in the observation group had significantly shorter in-bed activity time and out-of-bed activity time, average hospitalization time, and significantly lower total treatment costs, with statistically significant differences between the two groups (*P* < 0.05). Details are shown in Table 1.

**Table 1:** Comparison of time of in-bed and under-bed activities, hospital stay, and total cost of treatment between the two groups (*x̄* ± *s*)

Group	Number of cases (*n*)	Time of in-bed activities (h)	Time od under-bed activities (days)	Average hospitalization time (days)	Total cost of treatment (10k)
Control group	60	12.32 ± 3.45	5.24 ± 1.36	17.25 ± 4.28	3.18 ± 0.64
Observation group	60	6.85 ± 2.27	3.82 ± 0.94	10.67 ± 2.93	2.52 ± 0.32
*t* value		10.260	6.653	9.827	7.145
*P* value		0.001	0.001	0.001	0.001

### Comparison of incidence of complications within 4 weeks after operation between the two groups

**Fig. 1:**
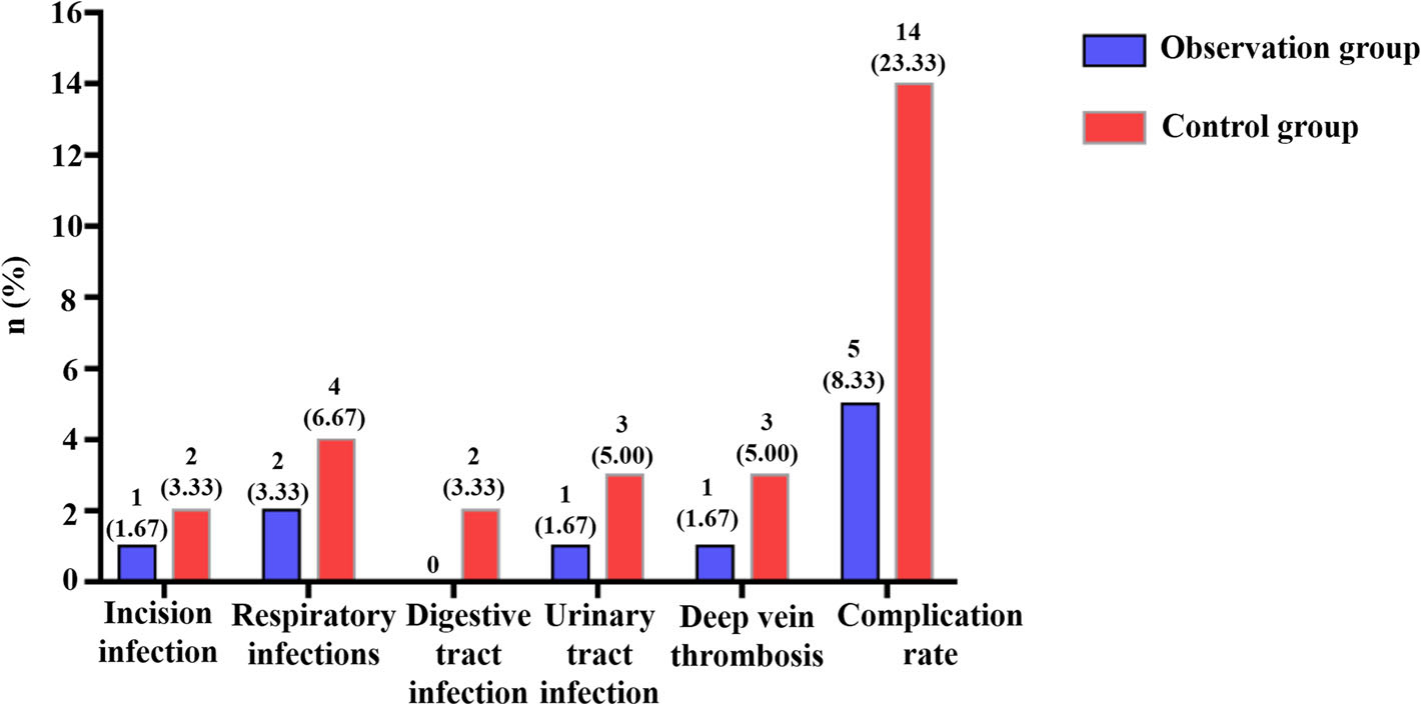
Comparison of complication rates between the observation group and the control group within 4 weeks after surgery

Within 4 weeks after the operation, 1 case of incision infection, 2 cases of respiratory system infection, 1 case of urinary tract infection, and 1 case of deep vein thrombosis occurred in the observation group. In the control group, there were 2 cases of incision infection, 4 cases of respiratory system infection, 2 cases of digestive tract infection, 3 cases of urinary tract infection, and 3 cases of deep vein thrombosis. The incidence of complications in the observation group (8.33%) was significantly lower than that in the control group (23.33%), and the difference between the groups was statistically significant (*x*^2^ = 8.443, *P* = 0.004). Details are shown in Fig. 1.

### Comparison of the recovery of joint function within 1 year after operation between the two groups

**Fig. 2:**
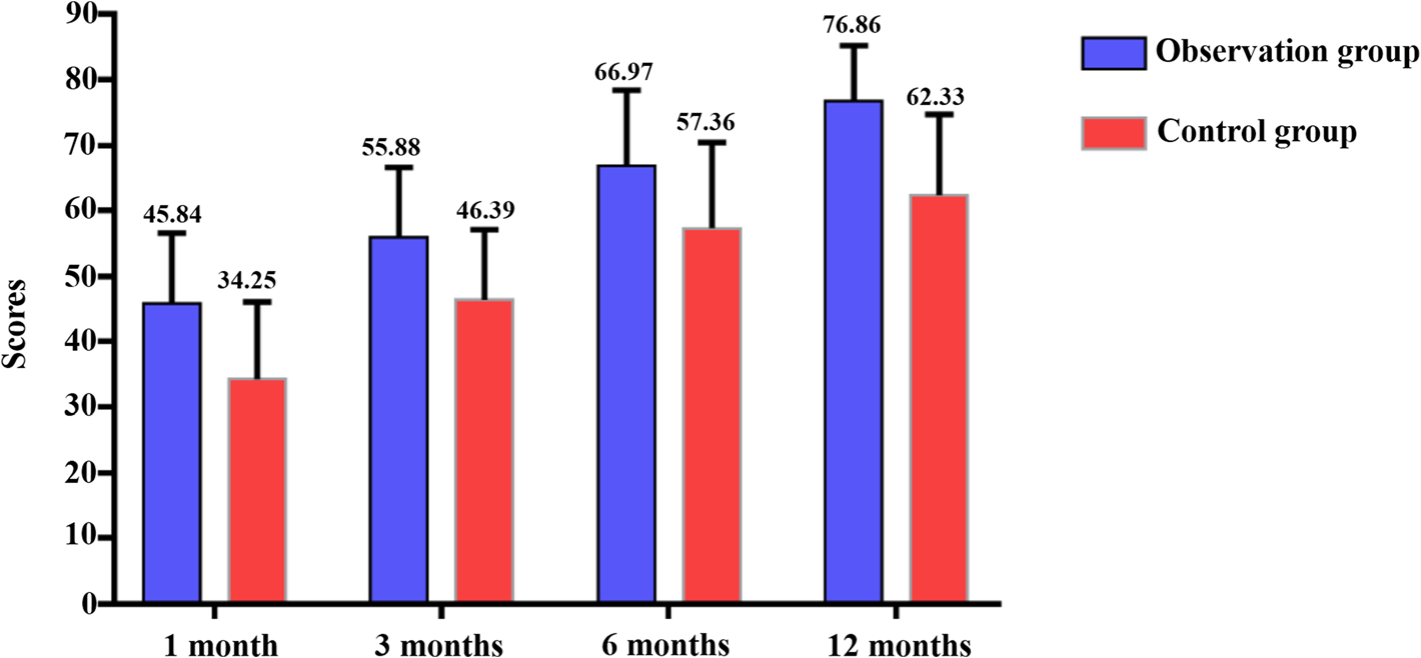
Comparison of joint function recovery scores between the observation group and the control group within 1 year after surgery

During the follow-up, the recovery scores of joint function in the observation group at 1 month, 3 months, 6 months, and 12 months after the operation were all better than those in the control group, and the recovery rate of joint function within 1 year after the operation was higher than that in the control group, with statistically significant difference (*P* < 0.05). Details are shown in Table 2 and Fig. 2.

**Table 2:** Comparison of the recovery of joint function within 1 year after operation between the two groups

Group	Number of cases (*n*)	Recovery score of postoperative joint function (*x̄* ± *s*)				Excellent and good rate of functional recovery within 1 year after operation [*n* (%)]
		1 month	3 months	6 months	12 months	
Control group	60	34.25 ± 5.63	46.39 ± 6.25	57.36 ± 7.24	62.33 ± 7.52	39 (65.00)
Observation group	60	45.84 ± 4.37	55.88 ± 5.32	66.97 ± 5.89	76.86 ± 6.03	52 (86.67)
*t*/*x*^2^ value		12.597	8.956	7.976	11.676	12.812
*P* value		0.001	0.001	0.001	0.001	0.001

### Comparison of clinical nursing satisfaction of patients in the two groups

The overall satisfaction rate of the two groups was statistically significant (*P* < 0.05). Details are shown in Table 3.

**Table 3:** Comparison of clinical nursing satisfaction of patients in the two groups

Group	Number of cases (*n*)	Very satisfied [*n* (%)]	Basically satisfied [*n* (%)]	Not satisfied [*n* (%)]	Overall satisfaction rate [*n* (%)]
Control group	60	19 (31.66)	28 (46.67)	13 (21.67)	47 (78.33)
Observation group	60	32 (53.33)	24 (40.00)	4 (6.67)	56 (93.33)
*x*^2^ value		9.608	0.906	9.250	9.250
*P* value		0.002	0.341	0.002	0.002

## Discussion

As a relatively new medical concept in recent years, the concept of accelerated rehabilitation surgery has been widely applied in the clinical nursing of surgical patients and achieved satisfactory nursing effect [7]. Applied to surgical care, the concept refers to the implementation of a series of evidence-based, high-quality nursing measures for surgical patients during the perioperative period. On one hand, by relieving the bad mood and psychology of the patients, it can reduce the traumatic stress of the patients; on the other hand, through the cooperation of medical staff and the orderly implementation of treatment and nursing plan, the operation can be promoted smoothly, so as to shorten the postoperative hospital stay and rehabilitation process [8]. At present, a large number of studies and investigations have shown that the clinical nursing mode based on the concept of accelerated rehabilitation surgery has achieved good results in the surgical treatment of gastric cancer, rectal cancer, primary liver cancer, uterine fibroids, and other diseases [9]. In orthopedic surgery, the risks during the operation are significantly increased due to the high dose of anesthesia, hunger state of patients, pain stimulation, and stimulation of operating instruments, etc., and the incidence of related complications is increased, thus seriously affecting postoperative recovery. Therefore, such patients have higher requirements for perioperative nursing care [10, 11]. To integrate the concept of accelerated rehabilitation surgery into the perioperative nursing of orthopedic surgery is of certain significance to the postoperative rehabilitation of patients.

Traumatic limb fractures are very common in orthopedic diseases, most of which are caused by traffic accidents or collisions, etc. Therefore, in clinical practice, surgery should be actively performed to reduce and fix fractures [12]. Patients with traumatic fractures usually worry about whether the operation can be carried out smoothly and are prone to have anxiety, tension, fear, and other adverse emotions and psychology before surgery. In addition, affected by the stimulation of trauma and surgical operation, the patient’s physiology is in a highly stressed state, which increases the risk of adverse reactions during the operation, and the probability of postoperative complications, and seriously hinders the recovery of postoperative limb joint function [13, 14]. In this study, the observation group of 60 patients with traumatic limb fractures implemented the concept nursing program of accelerated rehabilitation surgery during the perioperative period and implemented the nursing plan from the aspects of preoperative abstinence from drinking and fasting, psychological counseling, close cooperation during the operation, and early postoperative rehabilitation guidance and discharge guidance. The data results showed that the postoperative in-bed and out-of-bed activity time of patients and average hospital stay time were significantly shortened, and the total cost of treatment was also decreased, which was significantly different from the control group that implemented routine orthopedic nursing. The results show that the following the concept of nursing in rehabilitation surgery is helpful to promote the operation conducted smoothly, shorten the hospitalization time of patients, and save the treatment cost to some extent. Complications in the two groups were observed within 4 weeks after surgery, and the incidence of infection and thrombosis in the control group was higher than that in the observation group, suggesting that the nursing plan in the observation group could effectively prevent complications related to orthopedic surgery and improve the safety of patients’ treatment and rehabilitation. The reason for this result is mainly related to perioperative accelerated rehabilitation surgical nursing alleviates the physiological stress response of patients, and good body condition is the key to preventing adverse reactions and complications caused by intraoperative operational stimulation for surgical patients [15]. After 1 year of follow-up, the study found that the recovery score of limb and joint function in the observation group was better than that in the control group, and the recovery rate was better than that in the control group, indicating that the nursing concept of accelerated rehabilitation surgery has positive significance in improving postoperative functional recovery of patients. The overall satisfaction rate of the observation group was higher than that of the control group, mainly due to the improvement of perioperative nursing quality, the reduction of postoperative complications, the improvement of safety, the good nursing experience of the patients, and the satisfaction rate increased.

A previous study showed that postoperative lower extremity rehabilitation exercise can effectively accelerate patients’ health recovery from the oblique lumbar interbody fusion surgery and increase their satisfaction [16], which was similar with our results. Another study has shown that rehabilitation nursing combined with conservative treatment can shorten the healing period and improve functional recovery in elderly compression fracture patients [17].

There were also some limitations in this study. First, this is a single-center study; thus, it is lacking extensive representativeness. Second, the blind method has not been employed throughout the whole study. Third, we have included only patients with closed traumatic limb fractures. In addition, it would be much better if the patient sample size is larger.

In conclusion, the application of the nursing model of the concept of accelerated rehabilitation surgery in orthopedic surgery can significantly shorten the hospital stay, reduce the cost of treatment, improve the quality of postoperative rehabilitation of patients, promote the recovery of limb and joint function, and effectively prevent the occurrence of complications such as infection and thrombosis, increase patient satisfaction, and maintain clinical promotion value.

## Data Availability

The datasets used or analyzed during the current study are available from the corresponding author on reasonable request.
